# SVhawkeye: an ultra-fast software for user-friendly visualization of targeted structural fragments from BAM files

**DOI:** 10.3389/fgene.2024.1352443

**Published:** 2024-04-24

**Authors:** Yuhui Xiao, Tao Yu, Fan Liang, Tingping Hou

**Affiliations:** ^1^ The State Key Laboratory of Refractories and Metallurgy, Joint International Research Laboratory of Refractories and Metallurgy, Hubei Province Key Laboratory of Systems Science in Metallurgical Process, International Research Institute for Steel Technology, Collaborative Innovation Center for Advanced Steels, Wuhan University of Science and Technology, Wuhan, China; ^2^ School of Optoelectronic Materials and Technology, Jianghan University, Wuhan, China; ^3^ GrandOmics Biosciences, Wuhan, China

**Keywords:** structural variations, data visualization, genotyping, third-generation sequencing, whole-genome sequencing, python and R software

## Abstract

SVhawkeye is a novel visualization software created to rapidly extract essential structural information from third-generation sequencing data, such as data generated by PacBio or Oxford Nanopore Technologies. Its primary focus is on visualizing various structural variations commonly encountered in whole-genome sequencing (WGS) experiments, including deletions, insertions, duplications, inversions, and translocations. Additionally, SVhawkeye has the capability to display isoform structures obtained from iso-seq data and provides interval depth visualization for deducing local copy number variation (CNV). One noteworthy feature of SVhawkeye is its capacity to genotype structural variations, a critical function that enhances the accuracy of structural variant genotyping. SVhawkeye is an open-source software developed using Python and R languages, and it is freely accessible on GitHub (https://github.com/yywan0913/SVhawkeye).

## Introduction

The genetic variations in the human genome encompass a variety of categories, including numerical abnormalities of chromosomes, structural variation (SV) in chromosomes, copy number variation (CNV), single nucleotide variation (SNV), and insertion-deletion mutations (indels). Numerical abnormalities of chromosomes can result in conditions like trisomy syndrome, which can be identified through methods such as karyotyping or analyzing the depth distribution of all chromosomes (Santoro et al., 2020; Santoro et al., 2022).

SVs denote significant genomic alterations that typically span at least 50 base pairs (Duan et al., 2022). These genomic variants include inversions, balanced translocations, and genomic imbalances, which involve duplications, insertions, and deletions collectively referred to as DNA gains, losses, or rearrangements (Sudmant et al., 2015; Sedlazeck et al., 2018a). Whole-genome sequencing (WGS) from next-generation sequencing (NGS) or third-generation sequencing (TGS) data can detect these variants.

SVs are not only play an important role in gene expression (Chain and Feulner, 2014; Chiang et al., 2017) and phenotypic diversity (MacArthur et al., 2007; Perry et al., 2007; Jarvis et al., 2012; Kamberov et al., 2013). Numerous complex hereditary diseases, including autism (Hoischen et al., 2014; Dennis et al., 2017), cancer (Vogelstein et al., 2013; Zack et al., 2013), Alzheimer’s disease (Roses, 2016), and schizophrenia (Klar, 2004; Marshall et al., 2017), are known to originate from structural variations in the genome. Examples of such variations encompass translocations and large deletions, significantly contributing to both cancer and hereditary diseases (Abeysinghe et al., 2006). Gene inversions also play a role in certain conditions, such as Hemophilia A (Dai et al., 2021). Moreover, Short Tandem Repeats (STRs) (Masters et al., 2001) and Variable Number of Tandem Repeats (VNTRs) (Bakhtiari et al., 2021), specific types of structural variations, have been extensively studied in connection with repeat expansions. For instance, ATTCC repeat expansions have been associated with Parkinson’s disease (Schüle et al., 2017), and CAG expansions have been linked to Huntington’s disease (McColgan and Tabrizi, 2018).

SNVs are the most common type of genetic variation in humans (Katsonis et al., 2014). They play a crucial role in phenotypic diversity. RNA splicing (Wilkinson et al., 2020), a significant biological process in eukaryotic gene expression, is frequently detected through RNA-seq or isoform sequencing (iso-seq). This process results in the creation of numerous functional mRNAs carrying coding information. CNV (Wong et al., 2007; Fanciulli et al., 2010), on the other hand, refers to variations in the number of copies of specific DNA segments across different individuals’ genomes, resulting from duplications, deletions, or other alterations, often indicated by changes in read depth in WGS or whole exome sequencing (WES).

To effectively detect SVs from long reads, numerous software packages have been developed using genomic sequence data. These tools, including sniffles (Sedlazeck et al., 2018b), cuteSV (Jiang et al., 2020), pbsv (https://github.com/PacificBiosciences/pbsv), and svim (Heller and Vingron, 2019), provide valuable SV results but may still have limitations in accurately identifying specific target SVs. Detected SVs can contain inaccuracies or lack sufficient read support, requiring meticulous manual interpretation using Integrative Genomics Viewer (IGV) (Robinson et al., 2011; Thorvaldsdottir et al., 2013; Robinson et al., 2017; Robinson et al., 2020). This process is time-consuming and involves importing reference genomes and BAM files (https://www.ncbi.nlm.nih.gov/sra/docs/submitformats/), as well as constant manual adjustments to observe structure types and read mapping quality. Additionally, users may overlook SV types resulting from split-mapping.

Alternative tools such as bamsnap (Kwon et al., 2021) and svviz (Spies et al., 2015) provide automation, but are unable to handle long read data and can be slow (Table 1) and more detailed information may be needed to understand the presented SV structure (Figure 2B, Figure 5B). Samplot (Belyeu et al., 2021) offers a rapid overview of mutation structure but may obscure essential read information, making it difficult to discern precise genotyping details and missing nearby structural information. Even inaccurate SV length may occur (Figure 3C).

**TABLE 1:** Feature comparisons of currently available SV plot tools.

Software	Variation type	Variation type judgment	Input format	Speed (second/sv)	Visual information reading	SV genotyping/recall	Supports long reads
**SVhawkeye**	Fullly support^a^	auto	bam + vcf/bed	3.29	obvious^d^	yes	yes
**Samplot**	Part^b^	specify	bam + vcf/pos	2.41	deliberative^e^	no	yes
**IGV**	—	no	bam + pos	—	—	no	yes
**IGVScreenshot**	—	no	bam + bed	22.36	complex^f^	no	yes
**Bamsnap**	—	no	bam + vcf/bed/pos	—	complex	no	no
**Svviz2**	part^c^	specify	bam + vcf/bcf	31.03	complex	no	no
**Svviz**	part^c^	specify	bam + pos	35.55	complex	no	no

^a^ DEL/INS/INV/DUP/TRA/SNV/InDel/CNV, e.g.

^b^ DEL/INS/INV/DUP.

^c^ DEL/INS/INV/TRA.

^d^ Obvious: It is possible to simultaneously observe factors such as SV, type; SV, length, and SV, supported reads. (Figure 2; Figure 3; Figure 4; Figure 5; Figure 6).

^e^ deliberative: Missing partial information for SV, e.g., missing number of variant reads and even possible inaccurate variant length (Figure 3).

^f^ Complex: More information may be needed to confirm the displayed SV, type. (Figure 2; Figure 4; Figure 5).

In this paper, we introduce SVhawkeye, a novel software that addresses these aforementioned shortcomings. SVhawkeye offers a comprehensive suite of detection and visualization tools for SV curation. It enables the rapid generation of multiple SV graphs from VCF files simultaneously. SVhawkeye meticulously reviews and interprets each read, highlighting those that support target SVs. It can help quickly screen for pathogenic variants in clinical samples detected through third-generation sequencing. Moreover, SVhawkeye has the capability to concurrently exhibit various samples, encompassing family and population samples. It also accommodates the visualization of other genomic structural types like SNVs, RNA splicing, and CNV.

## Methods

**FIGURE 1:**
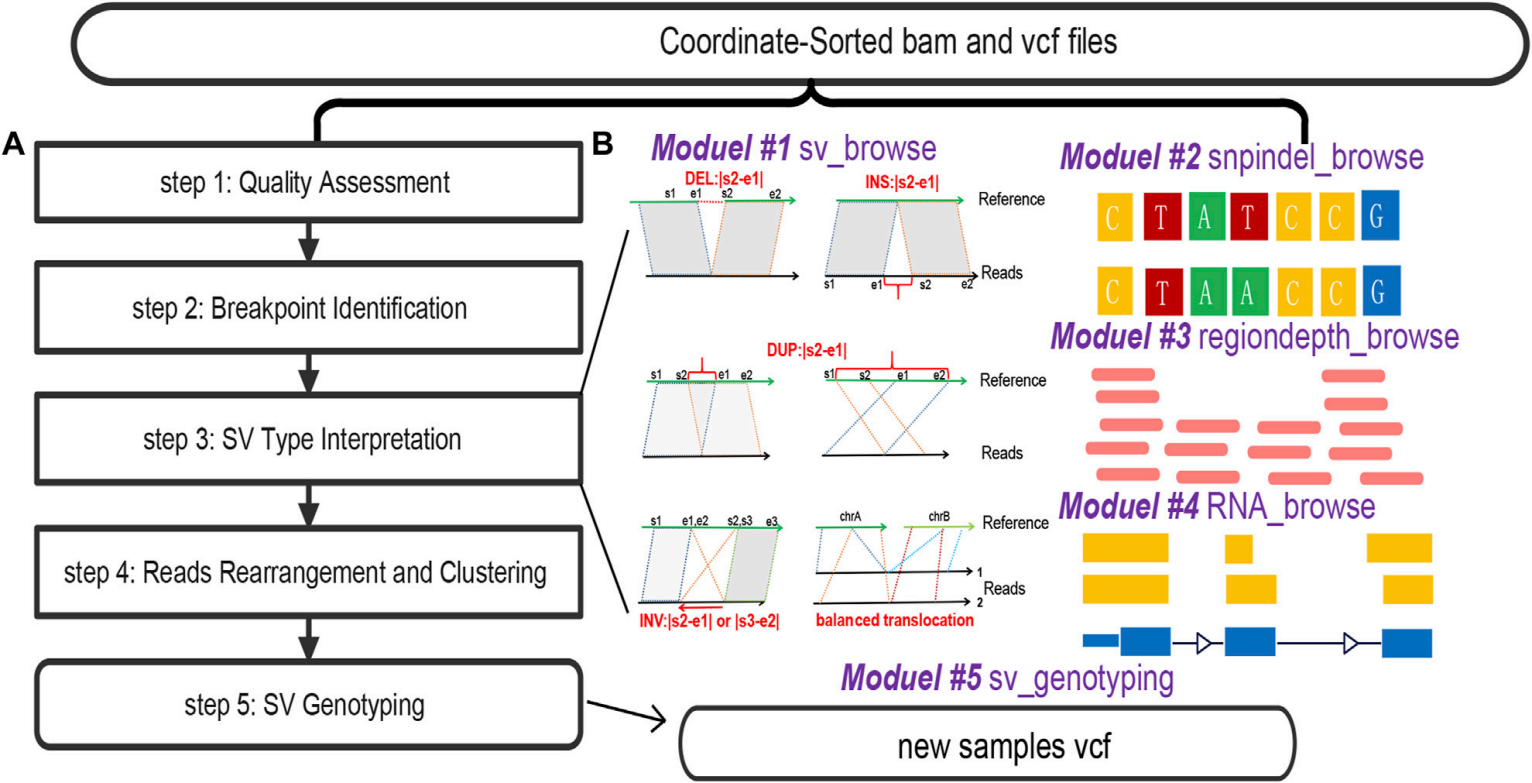
The overall workflow of SVhawkeye. **(A)** Data processing of SVhawkeye. **(B)** The SVhawkeye tool provides five modules, the first four modules can be used independently to graphically display gene variation information, and the last module can be used for SV genotyping. The input file displays that all modules require BAM file(s) and coordinate information for use.

SVhawkeye undergoes several pre-processing steps to prepare data for visualization (Figure 1A).

Step 1) Quality Assessment: Initially, SVhawkeye assesses the validity and quality of mapped reads, filtering out reads with insufficient evidence, Factors considered include low quality scores (default: mean reads quality ≥ Q20), low sequence identity (default: mapping identity ≥60%), and short mapping lengths (default: remove reads with mapping length less than 100 bp).
Step 2) Breakpoint Identification: SVhawkeye identifies breakpoints for each read based on cigar-mapping and split-mapping, utilizing the pysam package (https://pysam.readthedocs.io/en/stable/) to read the BAM file. The cigar information is utilized to identify possible deletions, insertions, and soft clipping events. Split-reads are employed to gather breakpoint data, serving as a foundation for interpreting the SV sequence.
Step 3) SV Type Interpretation: SVhawkeye interprets SV types using mapping coordinate information, amalgamating the breakpoint data from all reads and predicting SV characteristics through common SV types (Abel et al., 2020) and a well-designed algorithm. This relevant breakpoint information is subsequently recorded. In this step, all reads are recorded after comparison with the reference in various types, which can be reference reads, deletions, insertions, duplications, inversions or translocation reads, along with the corresponding breakpoint information.
Step 4) Reads Rearrangement and Clustering: To visualize SV structures with significant intervals, SVhawkeye utilizes a greedy non-overlapping interval clustering algorithm (https://labuladong.gitbook.io/algo-en/i.-dynamic-programming/intervalscheduling). This algorithm automatically arranges and assigns reads to each row in the graph, ensuring a visually appealing representation of SVs based on the previously acquired breakpoint information. Finally, plot these arranged coordinate information using the R language.
Step 5) SV Genotyping: SVhawkeye ascertains SV genotypes by scrutinizing the breakpoint information of reads that corroborate the specific SV within the interval relative to the target SV interval. This procedure entails excluding reads with coverage below 0.5 and breakpoint position disparities surpassing 1000, then removing reads with effective comparison length less than 100bp in this region. After reordering the reads in the step 4, the depth value also becomes more reasonable. Following this, SVhawkeye consolidates and computes the allele frequency (AF) value. A genotype of 0/1 is assigned when the AF value exceeds 0.3, while it is designated as 1/1 if the AF value surpasses 0.8.

### Workflow of SVhawkeye

The main ‘hawkeye.py’ function comprises five subroutines: ‘sv_browse,’ ‘snpindel_browse,’ ‘rna_browse,’ ‘regiondepth_browse,’ and ‘sv_genotyping.’ All of these subroutines require input in the form of BAM files and interval information, such as BED/VCF files or interval strings (Figure 1B).

### Moduel #1 sv_browse

The ‘hawkeye.py sv_browse’ command is used to visualize structural variants within a specified interval, sourced from either a BED or VCF file. Firstly, the graphical layout and details closely resemble those of the IGV software. ‘sv_browse’ offers the advantage of automatically identifying SV types from cigar-mapping and split-mapping reads. Furthermore, this software provides support for multiple sample files and facilitates the batch plotting of multiple intervals. Additionally, it demonstrates proficiency in presenting extensive intervals in a user-friendly fashion. It intelligently selects reads for display when confronted with an excessive quantity of fragments. This approach enables users to quickly assess the reliability of the target variant for subsequent analysis.

The using method of sv_browse command is as follows:

hawkeye.py sv_browse -i $bam -b $input.vcf -f vcf --thread $cpu -o $outdir --genome hg19 --quanlty 20 --sv_min_length 50 -F png

or

hawkeye.py sv_browse -i $bam -b $input.bed -f bed --thread $cpu -o $outdir --genome hg38 --quanlty 20 --sv_min_length 50 -F pdf

### Moduel #2 snpindel_browse

The ‘hawkeye.py snpindel_browse’ command is utilized to visualize SNVs or indel structures from sequencing data. Its usage closely mirrors that of “sv_browse,” and users have the option to specify a reference FASTA file using “-r $ref.fa” to display reference bases. This feature is very user-friendly for intervals less than 200bp.

### Moduel #3 regiondepth_browse

The “hawkeye.py regiondepth_browse” command is utilized to quickly visualize the depth of a region within a specific segment of the genome, especially in areas where deletions or duplications are present.

The using method of regiondepth_browse command is as follows:

hawkeye.py regiondepth_browse -i $bam -o $outdir -r ${chrom:start-end}

### Moduel #4 rna_browse

The “hawkeye.py rna_browse” command is used to visualize isoform structures obtained from RNA-seq or iso-seq data. Users need to provide a BED file containing the target interval and a GenePred (Hubisz and Siepel, 2023) file if the input species is not human.

The using method of rna_browse command is as follows:

hawkeye.py rna_browse -i $bam -b $inputbed -g $genome -t $cpu -o $outdir --genepred genome.genePred.gz

### Moduel #5 sv_genotyping

The “hawkeye.py sv_genotyping” command is employed to swiftly perform genotyping of structural variants using an accurate force-calling method.

The using method of sv_genotyping command is as follows:

hawkeye.py sv_genotyping -i $bam -b $vcf -f vcf -o $outdir -t $cpu

## Results

Table 1 presents a comprehensive comparison between SVhawkeye and commonly used SV plotting tools currently available. SVhawkeye boasts the following advantages.

**FIGURE 2:**
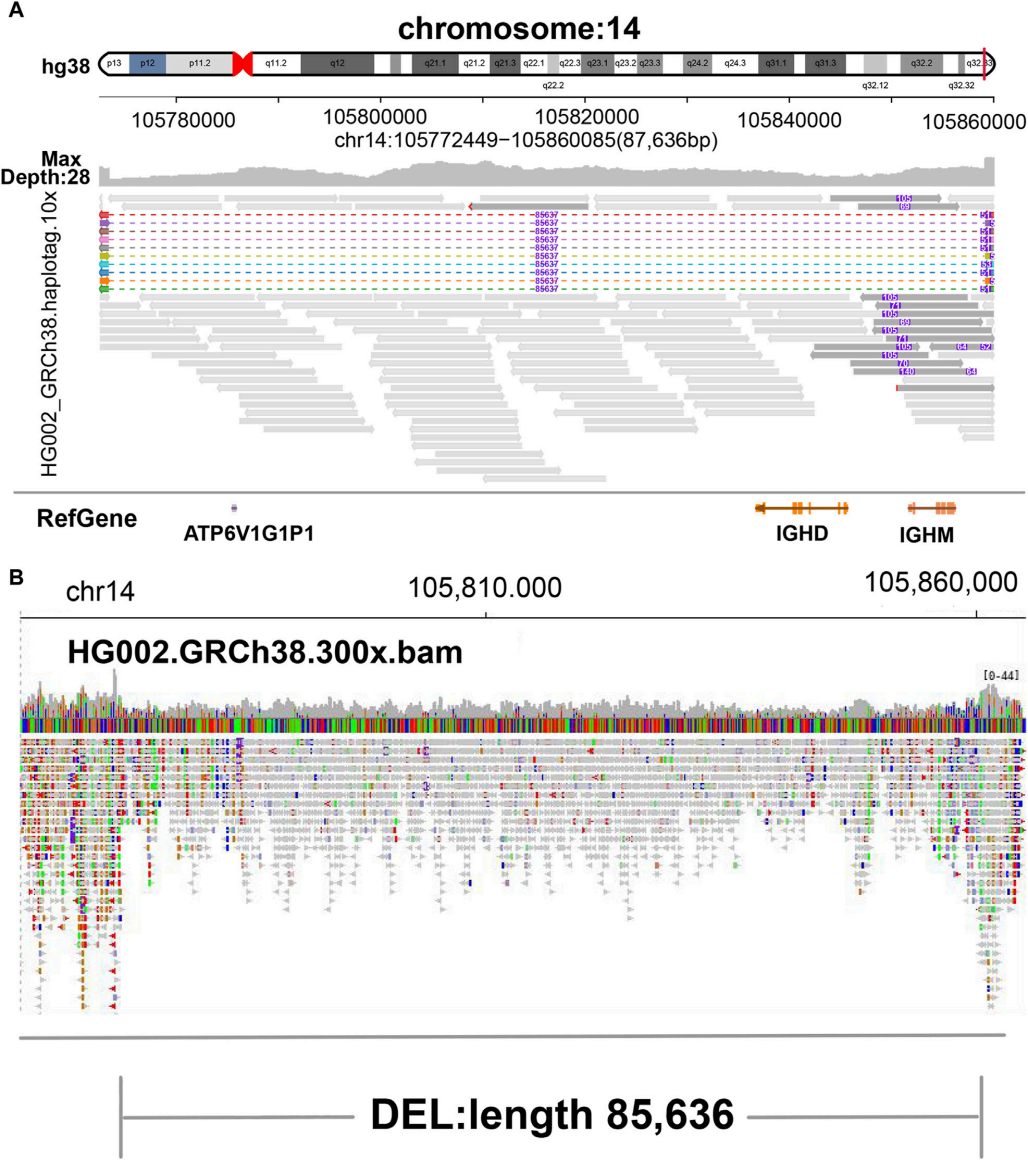
Display of deletion. **(A)** SVhawkeye. When reads are split-mapping, the colored dotted lines represent gaps, reads are aligned to both ends, and the purple number in the middle indicates the deletion length. When cigar-mapping, reads are linked by solid line. **(B)** Bamsnap.

**FIGURE 3:**
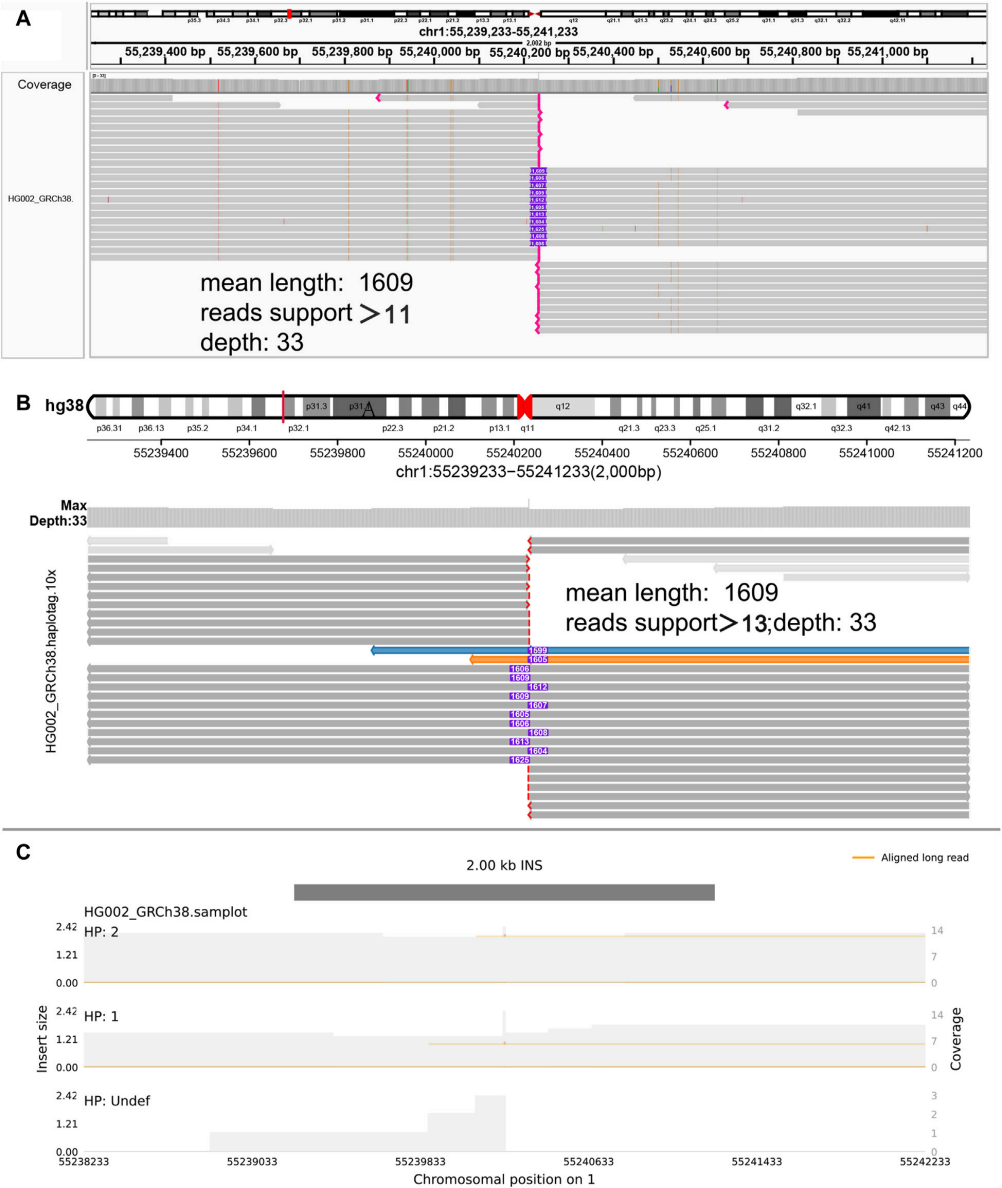
Display of insertion. **(A)** IGV. **(B)** SVhawkeye. When reads are split-mapping, they would be colored, and cigar-mapping would not be. The purple number in the middle indicates the insertion length. The red breakpoint at the end indicates soft-clipping which indicates that the reads may be too short to display an incomplete insert frequently. **(C)** Samplot.

**FIGURE 4:**
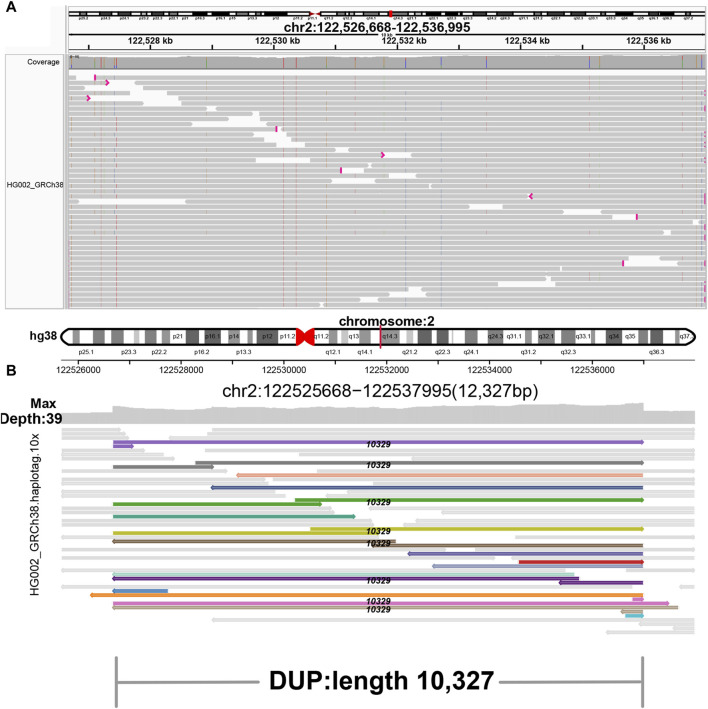
Display of duplication. **(A)** IGV. **(B)** SVhawkeye. Split-mapping reads are colored, and the black number in the middle of the breakpoint indicates the length of duplication.

**FIGURE 5:**
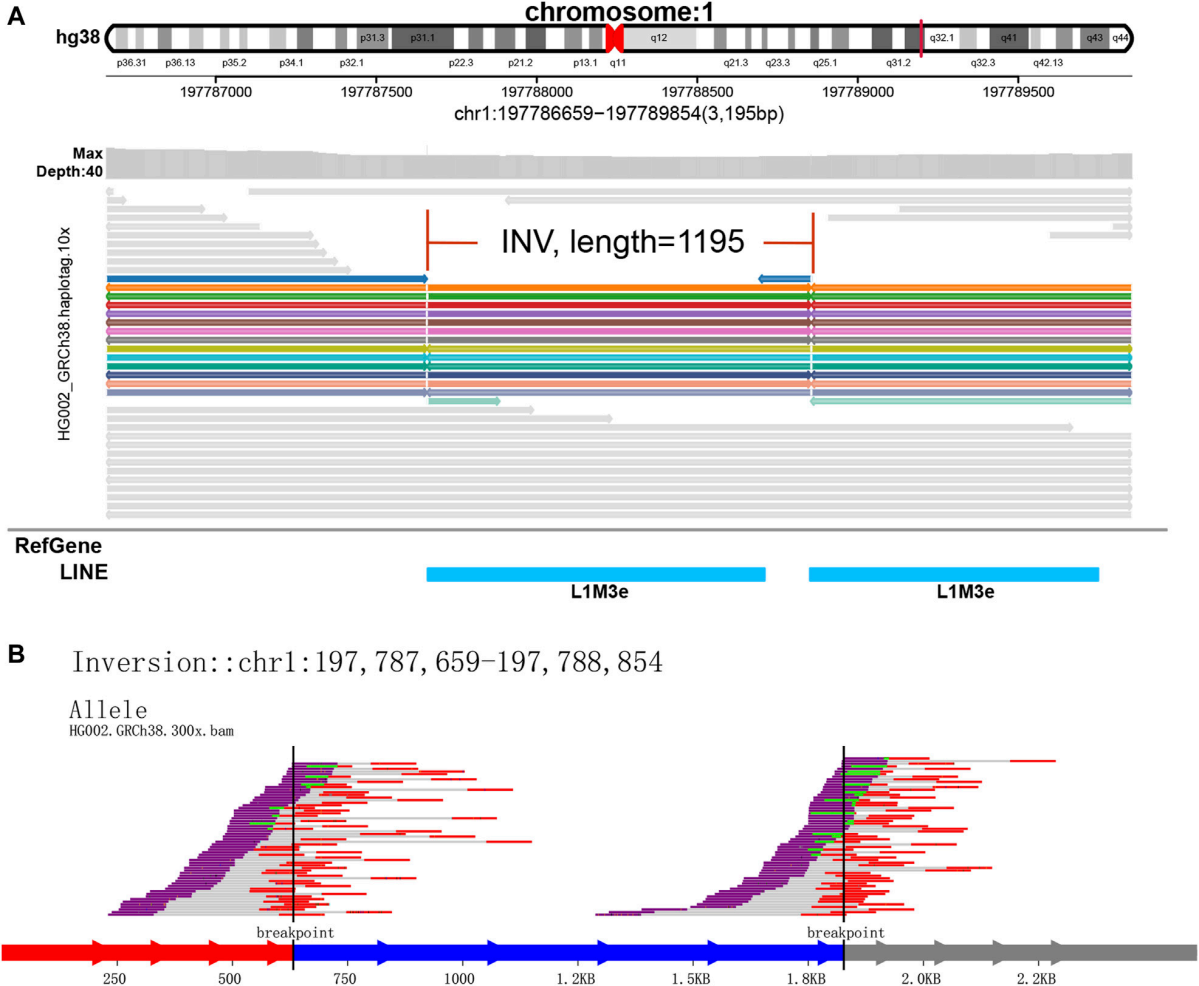
Display of inversion. It is caused by two breaks in the same chromosome, and the resulting pieces are reversed 180° and reconnected. **(A)** SVhawkeye. **(B)** Svviz

**FIGURE 6:**
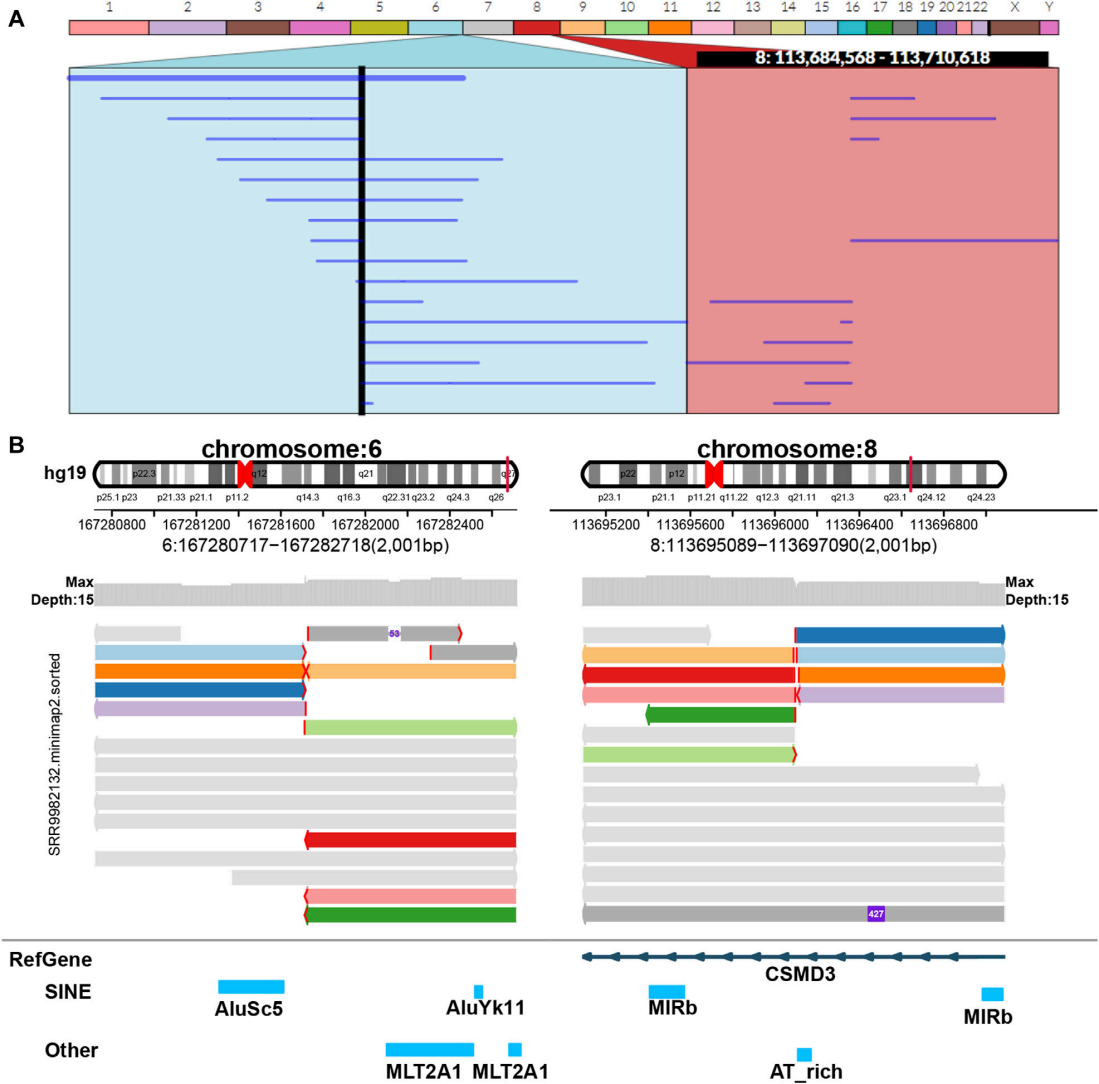
Display of balanced translocation. Two chromosomes from different sources are broken and then reconnected with each other to form two rearranged chromosomes in structure. The example data of balanced translocation are from NCBI (PRJNA559962) (Hu et al., 2020) and select the sample of SRR9982132. **(A)** Ribbon. **(B)** SVhawkeye.

1. Easy to Get Started: SVhawkeye is not required to install on personal computer, and includes an efficient matching annotation database. Users can operate it with a friendly one-click command-line operations, and only requires input the bam(s) and coordinate file (vcf/bed) (refer to Table 1 Input format column).
2. Diversification: SVhawkeye supports multi-threading capabilities, and has the ability to display and compare multiple samples simultaneously.
3. Support for Displaying Very Large Intervals: SVhawkeye excels in presenting data across extensive intervals. It organizes reads for an orderly arrangement and provides depth information simultaneously (Figure 2A). When handling an abundance of fragments, SVhawkeye intelligently selects a subset of reads for display, ensuring efficient visualization.
4. Automatic Variation Type Identification: In contrast to tools like Samplot and Svviz that require specifying SV types. SVhawkeye automatically identifies SV types based on coordinate information. It can highlight split-mapping reads and determined all SV types of reads within the interval, and marking their lengths numerically. (refer to Table 1 variation type judgment column).
5. Support for Displaying All Variation Types: SVhawkeye stands out by providing essential functionality for displaying all variation types, including deletion (Figure 2), insertion (Figure 3), duplication (Figure 4), inversion (Figure 5), balanced translocations (Figure 6) etc. This feature distinguishes it from other tools lacking this capability. (refer to Table 1 variation type column).
6. Speediness: The speed of SVhawkeye is at the forefront of ensuring more information and accuracy. The average drawing time per SV on Ubantu 18.04 LTS is approximately 3.29 s, only about 1 s slower than the Samplot tool (refer to Table 1 Speed column). On this basis, we also tested the performance of SVhawkeye on other aspects, the memory it occupies is related to the length and depth of the interval, usually not exceeding 1 g (RAM), which can be satisfied by a personal computer (refer to Table 2).
7. SV Genotyping: Due to SVhawkeye’s ability to determine the SV type for each read, SV genotyping is one of its strengths. However, SVhawkeye is better suited for long read sequencing. (refer to Table 1 SV genotyping and Supports long reads columns).

**TABLE 2:** Drawing efficiency of 1000 randomly selected SVs.

SVTYPE	SV counts	Max Len (/bp)	Threads	Max Memory (/M)	Run time (s)	Average time (/SV/thread)^a (s)^
DEL	437	61,031	4	15.2	26m19.34	3.61
INS	516	5,279	4	9.8	25m20.07	2.95
INV	47	11,660	4	11.4	3m12.27	4.09
ALL	1,000	61,031	4	15.2	54m51.68	3.29

^a^ Use Ubuntu 18.04 LTS, in DELL, Latitude 3510.

In our visualization results example, all the data is obtained from Genome in a Bottle (GIAB) (Nature, 2015). Figure 2 shows a deletion variation with a length of more than 85 K bp. SVhawkeye can quickly identify the mutation type, mutation length and the number of reads Supplementary Material (Figure 2A). Although Bamsnap is also a fast IGV-drawing based screenshot, it may require more information to support the displayed structural types. As it is only applicable to short reads and paired-end reads, it can quickly browse depth distribution information for NGS data (Figure 2B). Figure 3 is a comparison chart of insertion variation with a length of about 1.6 K. IGV (Figure 3A) can display the cigar-mapped insertion in a friendly way, with the insertion length matching the length in SVhawkeye. However, it is not easy to visualise insertions caused by split-mapping reads present in soft-clipping (represented by red triangles at one or both ends of the reads). Insertion in IGV lacks support for two reads coloured by SVhawkeye (Figure 3B). The depth information is ignored in samplot (Figure 3C) and there is a situation of fuzzy SV length (2 K). Figure 4 shows a DUP mutation with a length of more than 10 Kbp. As described in Figure 3A, if the mutation region is too long, the split-mapping reads make it difficult for IGV to display the SV type (Figure 4A) and may need to be interpreted by manually concatenating the included soft-clipping reads. SVhawkeye does not require these complex manual operations (Figure 4B). In the INV mutation example, SVhawkeye clearly shows the change in read alignment strand caused by the inversion (Figure 5A). The Svviz plot is difficult to understand for a while, but its presentation style is worth learning (Figure 5B). Finally, in the TRA example, SVhawkeye achieved a unique display compared to the aforementioned software, supporting reads at both ends of the breakpoint of the two chromosomes undergoing balanced translocation (Figure 6B). Of course, the Ribbon software (Nattestad et al., 2021) also supports this type of display (Figure 6A), but it cannot use command line operations and batch operations, so no detailed comparison was made.

In conclusion, SVhawkeye has a speed no lower than other software, and many easily understood factors have been added to the graph results, including SV type, SV length and SV read support. It can display the SV type with highlighted reads and record the SV length numerically. Samplot is fast and the graphs are clear and concise, but it may miss some important factors of SV, such as SV allele frequency, and may even give unreliable SV length. After all, for clinical samples, many detailed features need to be considered. The only drawback of IGV, as a commonly used software in the field of bioinformatics, is that it requires cumbersome steps to import bam file and adjust the reads to achieve the purpose of displaying specific mutation information. If the interval is large, it is difficult to display in IGV. Fortunately, Bamsnap and Svviz are more suitable for next-generation sequencing and are good at quickly displaying alignment information for short reads intervals. (refer to Table1 Visual information reading column).

At the bottom of the structural diagram, gene annotations content has been added, including gene name, repeatmaker and genomicSuperDups database information (Wang et al., 2010), provide valuable insights into the annotation of the breakpoint location.

In addition, SVhawkeye distinguishes itself by incorporating features such as SNP, InDel, RNA splicing, and CNV visualization, which are not present in other tools. In the SNP and InDel diagram (Supplementary Figure S1), users can conveniently access information for each base within the reads and include details about the reference sequence beneath the reads. The RNA splicing diagram (Supplementary Figure S2) enables users to specify a gene interval for visualizing its isoform structure, detected from iso-seq or full-length single-cell transcriptome data. The CNV plot (Supplementary Figure S3) requires users to input an interval to display the depth distribution of reads, facilitating the identification of the coverage range of the target area.

**FIGURE 7:**
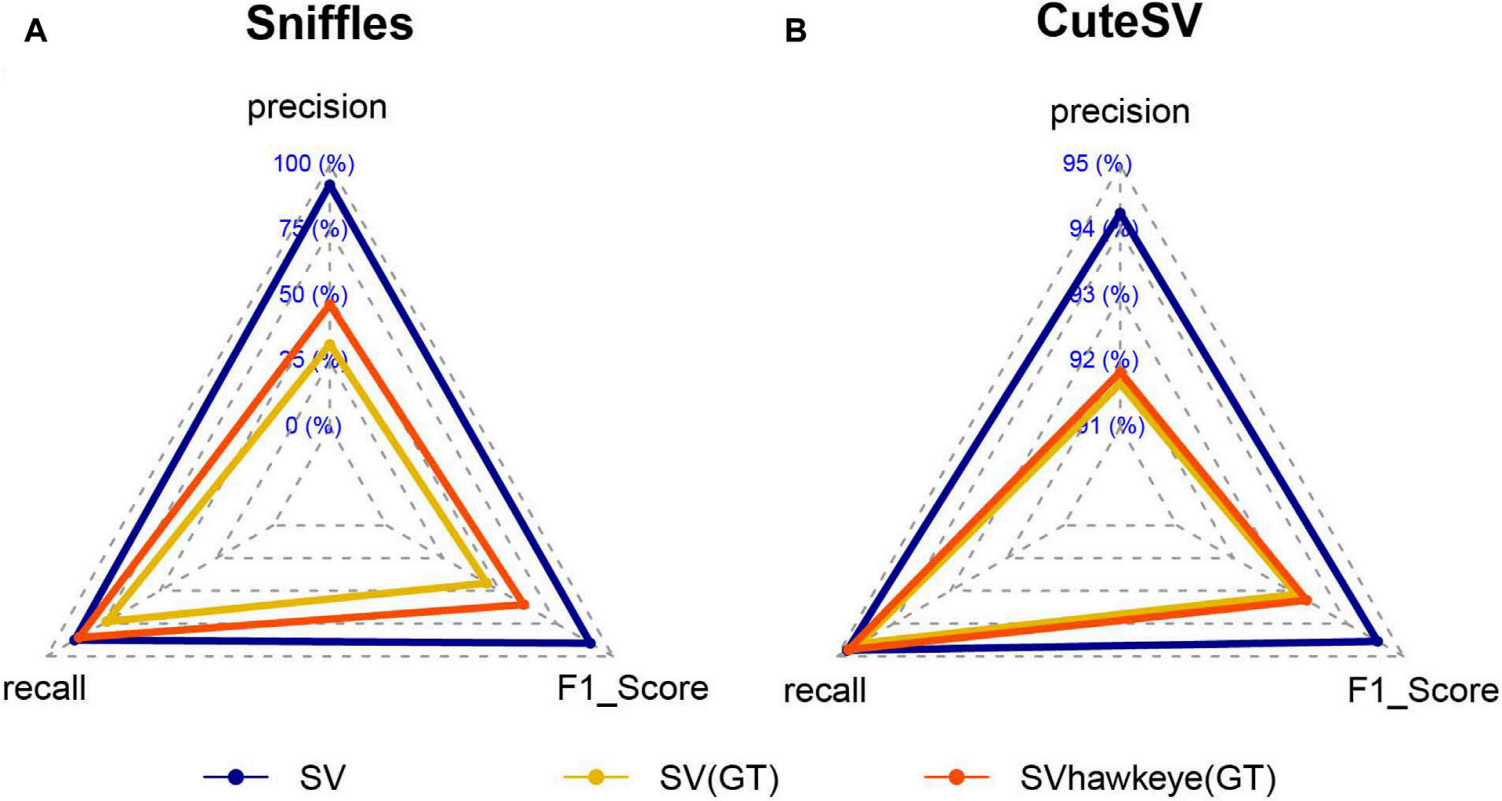
Comparison of accuracy of SV genotyping. **(A)** Genotyping from SVhawkeye compare to sniffles. **(B)** Genotyping from SVhawkeye compare to CuteSV.

The genotyping feature is an essential component of SVhawkeye, contributing significantly to the accuracy of SV genotyping (Figure 7). We utilized HG002 HiFi data for initial SV detection, employing tools such as sniffles and cute SV. Subsequently, we employed truvari (English et al., 2022) to compare SVhawkeye’s recall SV results with the initial SVs using the GIAB Tier1 v0.6 benchmark data (Zook et al., 2020). This analysis revealed a substantial improvement in genotype prediction accuracy, increasing from 31.6123% to 46.8814% for the sniffles results and from 90.8312% to 91.0554% for the cute SV results. Furthermore, the recall rate improved from 73.4472% to 85.6892% for the sniffles results and from 94.5167% to 94.75% for the cute SV results.

## Discussion and applications

In summary, the SVhawkeye software takes inspiration from the layout of IGV and aims to provide a swift, comprehensive visualization of structural variants. It automates the interpretation of read data and presents it in a meaningful and visually intuitive manner. This improved clarity and user-friendliness greatly streamline both the sequencing process and data interpretation, saving valuable time for biomedical researchers.

With SVhawkeye’s assistance, users can rapidly identify target regions across multiple disease samples, trio or pedigree samples, and more. For instance, SVhawkeye accurately detects balanced translocations, as demonstrated in various studies, including those referenced in PMC8804325 (Pei et al., 2022), benchmark structural variant research (Du et al., 2022), and population short tandem repeat counts, as verified in PMC9117641 (Liu et al., 2022). SVhawkeye is well-suited for detecting structural variants in clinical samples generated from PacBio or Oxford Nanopore sequencing. Of course, it is also suitable for other species and requires screening for more accurate variations, such as population variation analysis.

It is worth noting that while SVhawkeye offers valuable features, certain challenges in the sv_genotyping aspect, such as false-positive issues, remain unaddressed. These challenges include concerns such as read correction, breakpoint fragment realignment, and local assembly checking. Therefore, there is significant potential for future enhancements in the sv_genotyping component of SVhawkeye.

## Data Availability

The original contributions presented in the study are included in the article/Supplementary Material, further inquiries can be directed to the corresponding authors.
