# Supplementary figures and images for: SVhawkeye: an ultra-fast software for user-friendly visualization of targeted structural fragments from BAM files
Source: Front Genet. 2024 Apr 24;15:1352443. doi: 10.3389/fgene.2024.1352443 (PMC11076833; doi:10.3389/fgene.2024.1352443)

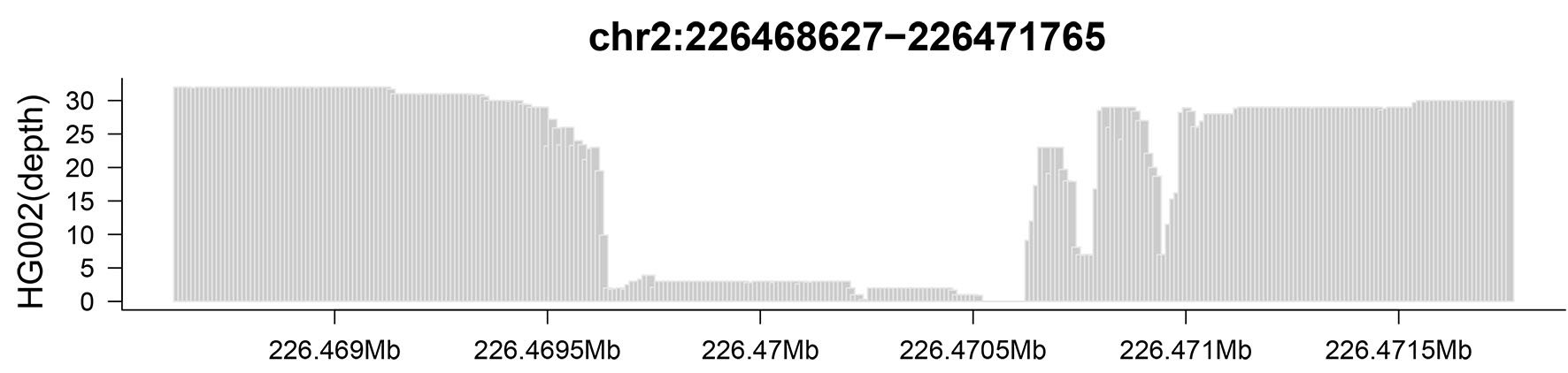

Supplement: Supplementary file 1 [file Image3.JPEG]

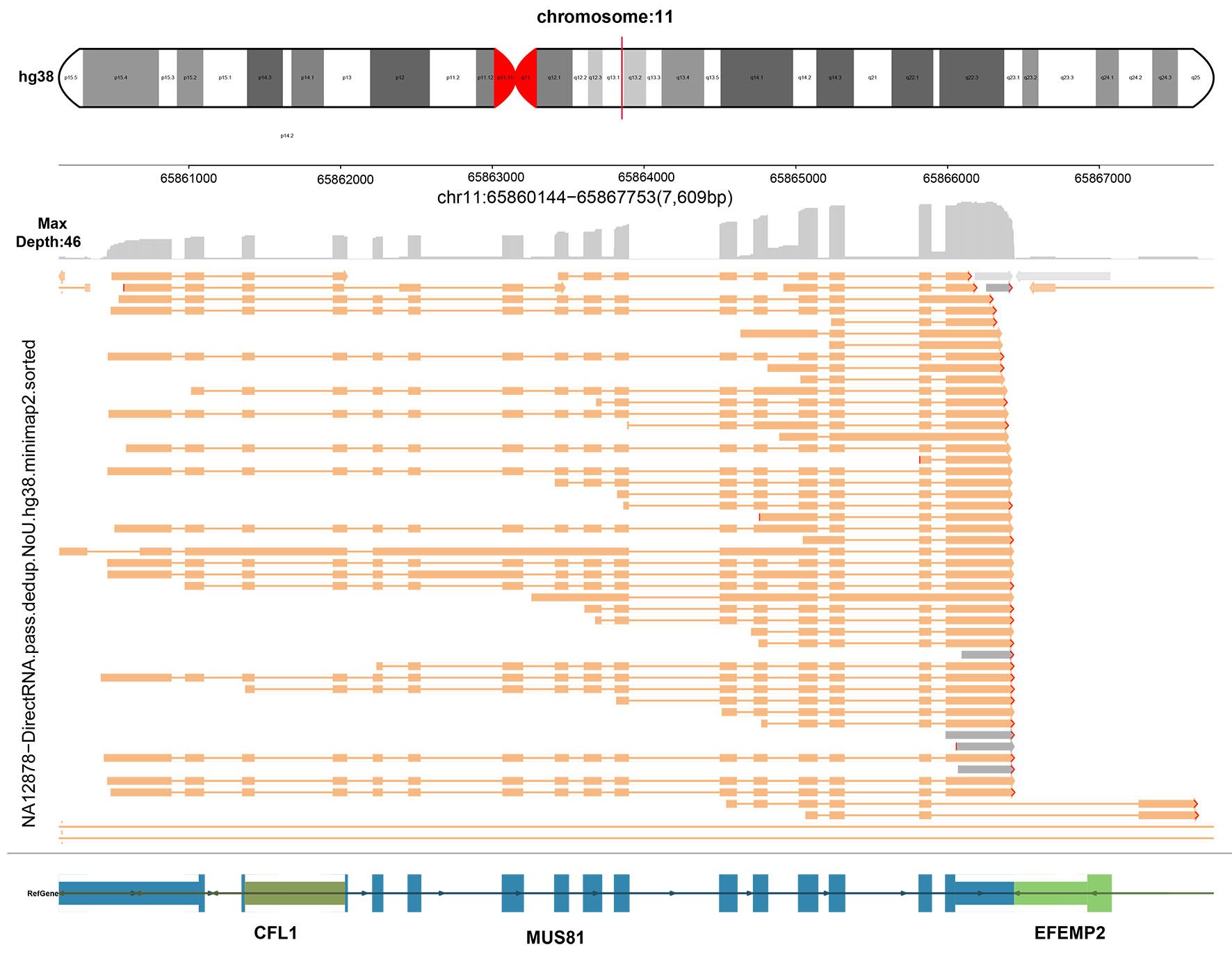

Supplement: Supplementary file 2 [file Image1.JPEG]

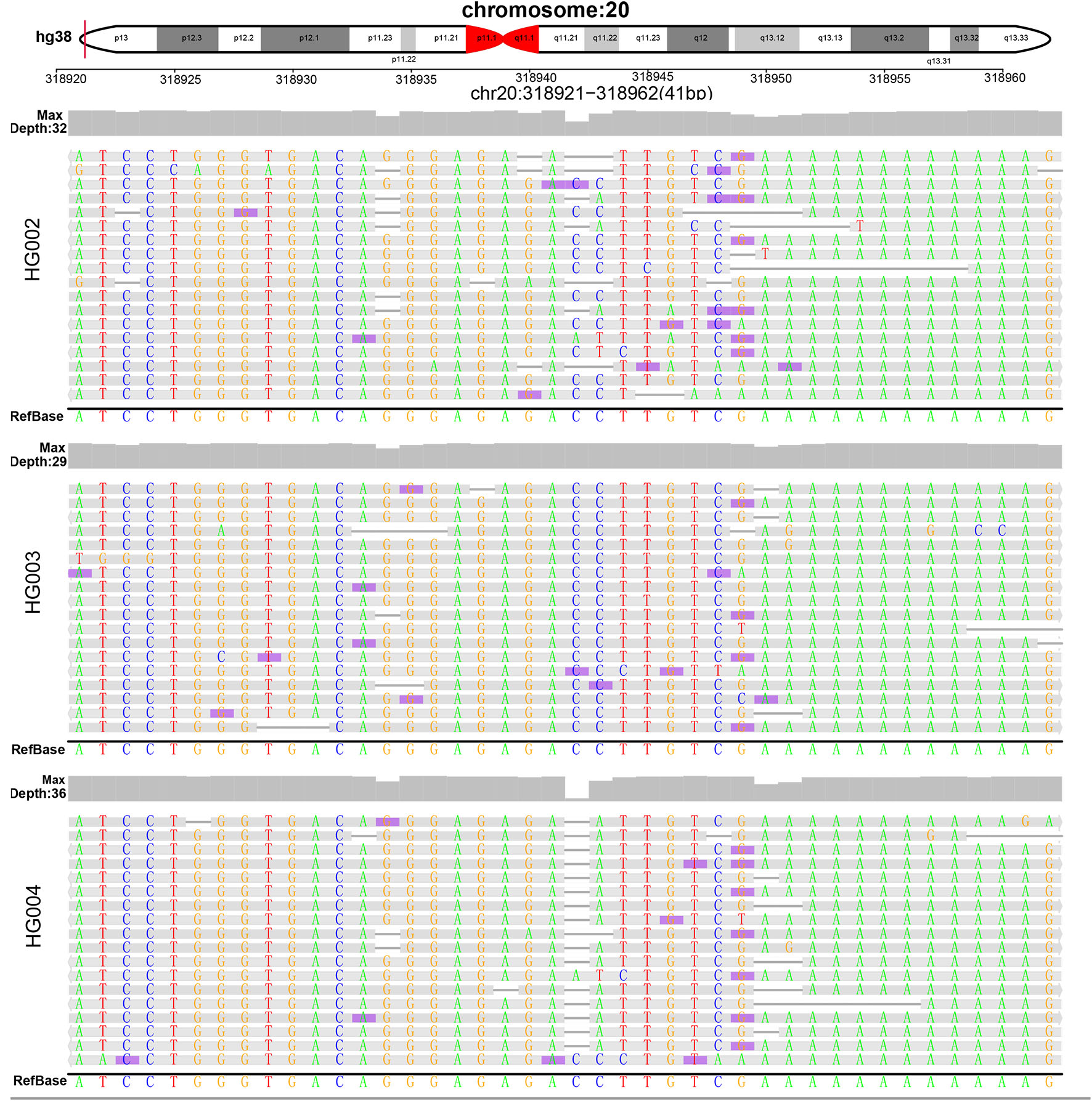

Supplement: Supplementary file 4 [file Image2.JPEG]
