# Supplementary material for: SVhawkeye: an ultra-fast software for user-friendly visualization of targeted structural fragments from BAM files
Source: Front Genet. 2024 Apr 24;15:1352443. doi: 10.3389/fgene.2024.1352443 (PMC11076833; doi:10.3389/fgene.2024.1352443)
Supplement: Supplementary file 5 [file DataSheet1.PDF]

Supplementary notes:RESULTS AND APPLICATIONS

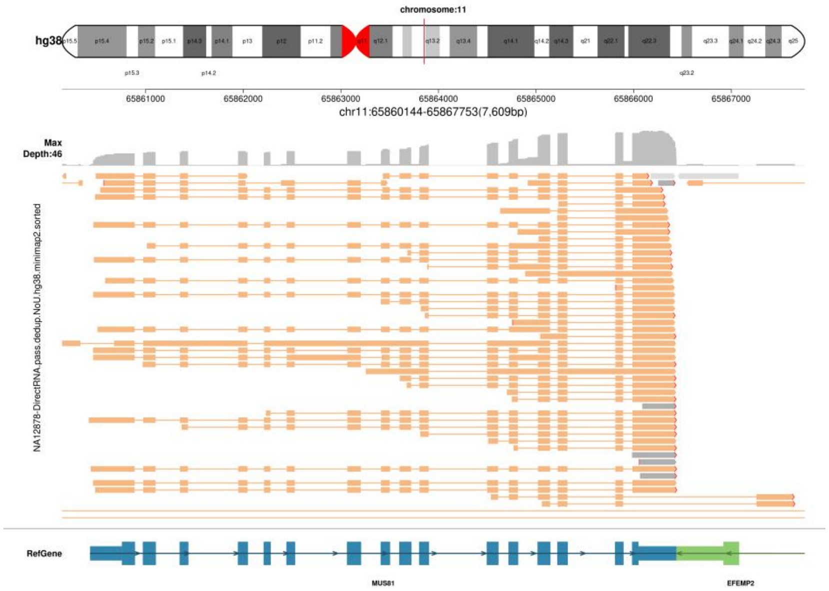

Figure S1. Display of isoform structure. The real data comes from GIAB, the NA12878 WGS sample(https://github.com/nanopore-wgs-consortium/NA12878). The alternative splicing or new isoform structure can be found from this function.

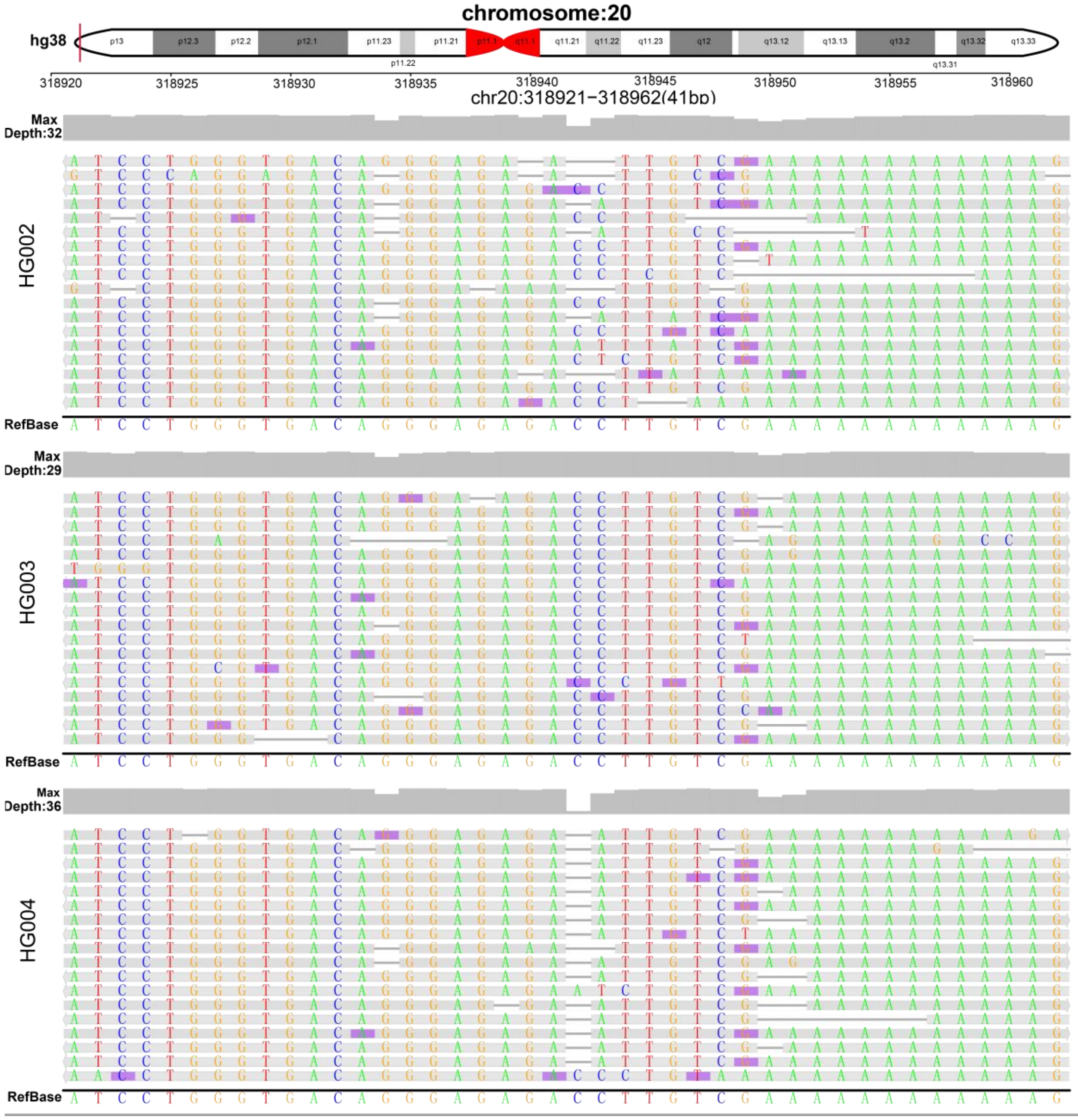

Figure S2. Display of SNVs and indel structure. The real data comes from GIAB, a benchmark developed by Zook et al. (ftp://ftptrace.ncbi.nlm.nih.gov/giab/ftp/data/AshkenazimTrio/).

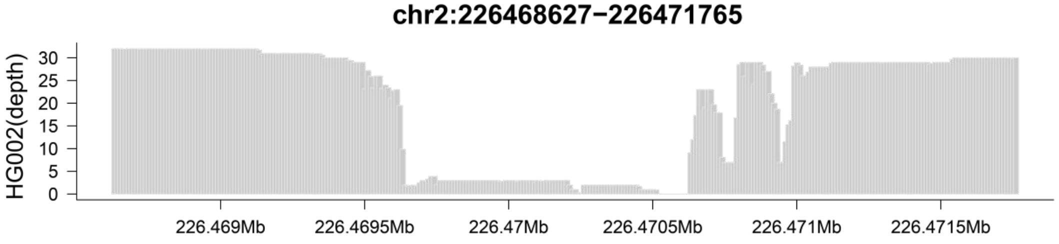

Figure S3. Display of depth distribution.
